# The Regulatory Network of FOXM1: Orchestrating Cancer Progression and Resistance to Therapy

**DOI:** 10.3390/ijms27125265

**Published:** 2026-06-10

**Authors:** Aleksei D. Korolev, Irina V. Bekbaeva, Polina V. Shnaider, Victoria O. Shender

**Affiliations:** 1 Lopukhin Federal Research and Clinical Center of Physical-Chemical Medicine of the Federal Medical and Biological Agency, 119435 Moscow, Russia; korolevad@my.msu.ru (A.D.K.); bekbaeva.iv@phystech.edu (I.V.B.); polya.shnaider@yandex.ru (P.V.S.); 2 Faculty of Biology, Lomonosov Moscow State University, 119991 Moscow, Russia; 3 Moscow Center for Advanced Studies, 123592 Moscow, Russia; 4 Shemyakin-Ovchinnikov Institute of Bioorganic Chemistry of the Russian Academy of Sciences, 117997 Moscow, Russia

**Keywords:** FOXM1, forkhead box M1, transcription factors, therapy resistance, interactome, intercellular communication, cancer

## Abstract

Therapy resistance remains a major obstacle to successful cancer treatment and is driven by complex interactions between tumor-intrinsic adaptive mechanisms and signals originating from the tumor microenvironment. Among the molecular regulators implicated in these processes, the transcription factor FOXM1 has emerged as a key mediator of DNA damage repair, cell cycle progression, and stress adaptation. Although FOXM1 has traditionally been studied as a regulator of intracellular signaling pathways, accumulating evidence suggests that its functions extend beyond canonical transcriptional control. In this review, we analyze current knowledge on the mechanisms regulating FOXM1 expression and activity and discuss how FOXM1 contributes to therapy resistance. We propose that FOXM1 should be viewed not merely as a regulator of individual oncogenic pathways but as a systems-level coordinator that integrates intracellular stress adaptation with microenvironment-driven resistance mechanisms. Particular attention is given to the FOXM1 interactome, complemented by an analysis of protein interaction data from BioGRID. We also discuss emerging evidence implicating FOXM1 in intercellular communication. To identify potential links between FOXM1 signaling and extracellular vesicle cargo, we analyzed the overlap between FOXM1 target genes and proteins identified in extracellular vesicle proteome databases. These emerging regulatory networks may represent previously underappreciated contributors to therapy resistance.

## 1. Introduction

Therapy resistance continues to undermine the efficacy of cancer treatment [1], contributing to treatment failure in advanced malignancies and posing a major challenge despite ongoing innovations in anticancer drug development. This phenomenon arises from both intrinsic tumor cell adaptations such as epigenetic reprogramming, enhanced DNA damage repair, and metabolic rewiring [2,3] and extrinsic factors within the tumor microenvironment, including immune suppression, stromal remodeling, and extracellular vesicle-mediated intercellular signaling [3,4,5]. Recent studies have highlighted how these processes converge to sustain cancer therapy resistance, bypass cell cycle checkpoints, and promote metastatic dissemination, underscoring the need for integrative molecular approaches to identify effective therapeutic targets [6,7].

**Figure 1:**
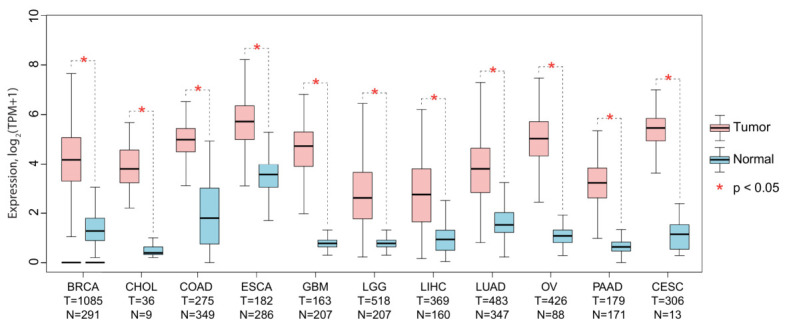
FOXM1 expression levels across different tumor types and normal tissues based on TCGA and GTEx datasets [15]. Tumor samples are denoted in pink, while normal samples are shown in blue. Dashed lines indicate the statistical comparisons between groups.

In this context, transcription factors that function as central regulatory hubs are of particular importance. Among them, the forkhead box transcription factor FOXM1 has emerged as a key coordinator of tumor cell responses to stress, with well-established roles in cell cycle progression, DNA damage repair, and resistance to genotoxic therapies across multiple cancer types [8]. FOXM1 is frequently overexpressed in malignant neoplasms (Figure 1) and is consistently associated with aggressive tumor behavior, unfavorable clinical outcomes [9], and resistance to a broad range of anticancer therapies, including platinum-based agents and taxanes [10,11,12]. These features have established FOXM1 as both a clinically relevant biomarker and an attractive therapeutic target [13,14].

Although FOXM1 has been extensively studied as a regulator of intracellular processes, its role extends beyond canonical transcriptional control. Recent evidence indicates that FOXM1 participates in complex protein–protein interaction networks, modulates multiple layers of gene expression, and contributes to intercellular communication within the tumor microenvironment. In particular, emerging data suggest that FOXM1 may influence not only the transcriptional landscape of tumor cells but also the composition of extracellular vesicles and therapy-induced secretomes, thereby facilitating the transfer of pro-survival signals between cells. Despite these advances, a comprehensive understanding of FOXM1 as an integrative node linking intracellular signaling, protein interaction networks, and intercellular communication in the context of therapy resistance remains incomplete. We propose that FOXM1 should be viewed not merely as a regulator of individual oncogenic pathways but as a systems-level coordinator that integrates intracellular stress adaptation with microenvironment-driven resistance mechanisms. A systematic analysis of these interconnected mechanisms may provide new insights into FOXM1-driven tumor adaptation and inform the development of more effective therapeutic strategies.

Compared with earlier reviews, the present review provides a broader systems-level perspective on FOXM1 biology by integrating evidence from the mechanisms regulating FOXM1 expression and activity, protein interaction networks, and intercellular communication within the tumor microenvironment. Particular emphasis is placed on recent findings related to the FOXM1 interactome and extracellular vesicle-mediated communication, which have received comparatively limited attention despite their potential importance in tumor adaptation and treatment failure. We also critically evaluate current FOXM1-targeting strategies and the major challenges that continue to hinder their clinical translation.

## 2. Structure of the FOXM1 Gene and Protein

FOXM1 is a member of the forkhead box (FOX) family of transcription factors, which are characterized by a highly conserved forkhead (FKH) DNA-binding domain [16]. This domain mediates sequence-specific interactions with target gene promoters and underlies the transcriptional regulatory functions of FOX proteins.

**Figure 2:**
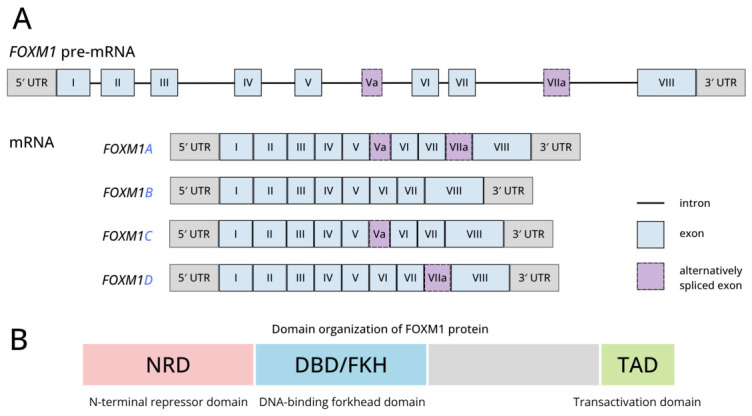
Structure of FOXM1. (**A**) Schematic repressentation of *FOXM1* pre-mRNA, comprising 10 exons (I–VIII), including the alternatively spliced exons Va and VIIa (purple), and the corresponding mRNAs of four *FOXM1* isoforms (*FOXM1A*, *FOXM1B*, *FOXM1C*, *FOXM1D*). (**B**) Domain structure of the FOXM1 protein: NRD, N-terminal repressive domain; DBD/FKH, DNA-binding forkhead domain; TAD, transactivation domain.

The *FOXM1* gene consists of 10 exons [16]. Through alternative splicing, it produces four mRNA isoforms: *FOXM1A*, *FOXM1B*, *FOXM1C*, and *FOXM1D*. These isoforms differ in the inclusion or exclusion of the alternatively spliced exons Va and VIIa: the *FOXM1A* isoform contains all exons, *FOXM1C* skips exon VIIa, *FOXM1D* omits exon Va, and *FOXM1B* lacks both Va and VIIa (Figure 2A). Functionally, FOXM1B [17], FOXM1C [18], and FOXM1D [19] act as transcriptional activators, whereas FOXM1A exhibits transcriptional repressor activity [20]. A recently discovered isoform, FOXM1s, further expands this diversity. Experimental studies have shown that FOXM1s is actively expressed in pancreatic cancer cells, where it promotes proliferation, migration, and invasion and increases the expression of stemness-associated genes [21].

It has been reported that the FOXM1C isoform predominates in normal cells and in several malignancies, including cholangiocarcinoma [18], ovarian cancer, and breast cancer [22]. At the same time, other studies have identified FOXM1B as the predominant isoform in lung cancer, breast cancer, cervical cancer, ovarian cancer, myeloid leukemia [17], and hepatocellular carcinoma [23]. Throughout this review, the term FOXM1 refers collectively to the FOXM1B and FOXM1C isoforms unless otherwise specified, as both are highly expressed in tumor cells and perform a similar function—activation of target gene transcription.

At the protein level, FOXM1 consists of three functional domains: an N-terminal repressive domain, a central forkhead DNA-binding domain, and a C-terminal transactivation domain (Figure 2B). The alternatively spliced exons Va and VIIa encode regions within the C-terminal transactivation domain [24]. Two intrinsically disordered regions, located between the DNA-binding domain and the C-terminal transactivation domain, play a critical role in the formation of FOXM1 transcriptional condensates at target gene promoters [25].

FOXM1 recognizes and binds specific DNA motifs within regulatory regions of its target genes. Similar to other forkhead transcription factors, it preferentially binds the consensus motif RYAAAYA (R = A/G; Y = C/T) [26], with a particularly high affinity for the C/TAAACA motif [27]. FOXM1 can also bind cooperatively to tandem DNA elements, such as the DIV2 site, which consists of two inversely oriented GTAAACA motifs, thereby enhancing transcriptional activation [28].

## 3. Mechanisms Regulating FOXM1 Expression and Activity

### 3.1. Regulation of FOXM1 Expression

*FOXM1* gene expression is tightly regulated at multiple regulatory levels, including transcriptional and post-transcriptional mechanisms, whereas its functional activity is primarily controlled by post-translational modifications.

**Figure 3:**
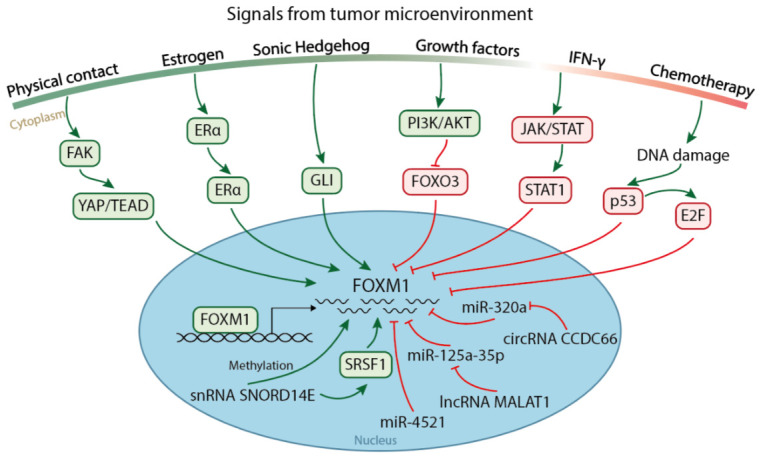
Intracellular pathways regulating *FOXM1* gene expression. Green arrows indicate positive regulation, whereas red arrows indicate inhibition.

Multiple signaling pathways converge to promote *FOXM1* transcription (Figure 3). Activation of growth factor receptors triggers the PI3K/AKT signaling pathway, which inactivates the FOXM1 repressor FOXO3, thereby promoting *FOXM1* expression [29]. The Sonic Hedgehog pathway increases *FOXM1* expression through the activation of GLI transcription factors [30]. Upon estrogen binding, the ERα receptor dissociates from the HSP90 chaperone complex and binds to the *FOXM1* promoter, leading to increased *FOXM1* expression [31]. In ovarian cancer cells, physical contact with cells of the tumor microenvironment activates focal adhesion kinase (FAK), resulting in the translocation of YAP to the nucleus [32]; TEAD transcription factors subsequently bind YAP and enhance *FOXM1* transcription [33].

Post-transcriptional regulation of FOXM1 is largely mediated by non-coding RNAs. Various microRNAs suppress *FOXM1* expression by binding to its 3′-untranslated region, thereby triggering mRNA degradation and repressing translation [34]. For example, miR-4521 suppresses *FOXM1* expression and induces tumor cell death in breast cancer [35] and medulloblastoma [36]. In turn, long non-coding RNAs and circular RNAs can compete with *FOXM1* mRNA for microRNA binding, thereby indirectly increasing *FOXM1* expression. For instance, in hepatocellular carcinoma cells, the long non-coding RNA *MALAT1* sequesters miR-125a-3p, which targets *FOXM1* mRNA [37], whereas the circular RNA circCCDC66 sponges miR-320a, thereby enhancing *FOXM1* expression in glioma cells [38]. The small nucleolar RNA *SNORD14E* recruits the splicing factor SRSF1, promoting the formation of the *FOXM1B* and *FOXM1C* isoforms. Additionally, *SNORD14E* methylates *FOXM1* mRNA, thereby increasing its stability [39]. Notably, FOXM1 also exhibits autoregulation: the FOXM1 protein binds to its own promoter and activates its transcription [40].

In addition to activating pathways, *FOXM1* is also subject to several negative regulatory mechanisms. Interferon-γ signaling activates the JAK/STAT pathway, leading to STAT1-mediated repression of *FOXM1* transcription [41]. Following DNA damage, the tumor suppressor p53 suppresses *FOXM1* transcription either directly or via E2F transcription factors [42]. FOXM1 is also regulated by autorepression, whereby the N-terminal repressive domain inhibits the C-terminal transactivation domain through hydrophobic interactions between specific motifs. This mechanism operates under basal conditions in proliferating cells during the G1 and S phases of the cell cycle [43].

### 3.2. Post-Translational Regulation of FOXM1

**Figure 4:**
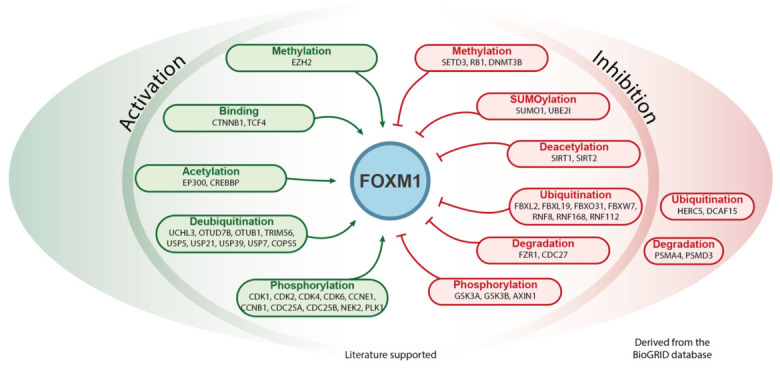
Functional landscape of the FOXM1 interactome regulating its stability and activity. Green arrows indicate positive regulation, whereas red arrows indicate inhibition. Additional putative FOXM1 interactors that may regulate FOXM1 stability were identified through analysis of the BioGRID database.

The activity, stability, and intracellular localization of FOXM1 depend on a wide range of post-translational modifications that dynamically fine-tune its function in a context-dependent manner. Although many FOXM1 protein–protein interactions have been characterized, the full spectrum of FOXM1-associated regulatory proteins remains incompletely understood. In addition to the well-characterized regulatory mechanisms described in the literature, we further expanded the landscape of FOXM1-associated regulatory proteins by integrating interactome data from the BioGRID database (Figure 4) [44]. The principal mechanisms governing the post-translational regulation of FOXM1 are discussed below.

#### 3.2.1. Ubiquitination and Degradation

FOXM1 stability is primarily controlled by the ubiquitin–proteasome system. Constitutively active SCF-family E3 ubiquitin ligases, including FBXW7, FBXO31, FBXL2, and FBXL19, promote FOXM1 degradation (Figure 4). During the cell cycle, FOXM1 levels are tightly regulated to prevent premature activation. Prior to mitotic entry, low FOXM1 levels are maintained by the SCF and CRL4 ubiquitin ligase complexes, with CRL4 likely recruiting FOXM1 via DCAF15 (Figure 4) [45,46]. At mitotic exit and during early G1 phase, the anaphase-promoting complex/cyclosome (APC/C) further regulates FOXM1 degradation through its components CDC27 and FZR1 (Figure 4) [47,48], thereby preventing premature FOXM1 accumulation in the absence of mitotic signals. In addition to constitutive ubiquitination, FOXM1 stability can be modulated in response to cellular stress. For instance, DNA damage induces the activity of E3 ubiquitin ligases such as RNF8, RNF168, and RNF112, leading to FOXM1 degradation and attenuation of its activity (Figure 4) [49,50].

#### 3.2.2. Deubiquitination

FOXM1 stability is further maintained by deubiquitinases belonging to the USP and OTU subfamilies (Figure 4), which prevent its proteasomal degradation and thereby promote malignant transformation [51,52,53]. The COPS5 protein, a regulator of CRL ubiquitin ligase complexes, also enhances FOXM1 stability through deubiquitination (Figure 4) [54,55]. In addition, ubiquitin-editing enzymes, which possess both ubiquitin ligase and deubiquitinase activities, contribute to FOXM1 regulation. For example, TRIM56 stabilizes FOXM1 by promoting its deubiquitination (Figure 4) [56,57].

#### 3.2.3. SUMOylation and Interaction with Proteasome Components

SUMOylation exerts isoform- and site-specific effects on FOXM1. SUMO-1 modification at distinct lysine residues, including K201, K218, K460, K478, and K495 in FOXM1C, and K463 as the major acceptor site in FOXM1B, promotes FOXM1 translocation to the cytoplasm and facilitates its APC/C-mediated proteasomal degradation (Figure 4) [58]. In contrast, SUMO-2 conjugation at multiple lysine residues distinct from the SUMO-1 modification sites disrupts the intramolecular interaction between the N-terminal repressive domain and the C-terminal transactivation domain, thereby preventing FOXM1 dimerization and enhancing its transcriptional activity [59]. Direct interactions between FOXM1 and proteasome components, including PSMA4 and PSMD3, have also been reported, suggesting a close functional link between FOXM1 activity and proteasomal regulation (Figure 4) [60].

#### 3.2.4. Phosphorylation

Among the most extensively characterized activators of FOXM1 are cyclin-dependent kinases (CDK1, CDK2, CDK4, and CDK6) and their associated cyclins B and E (Figure 4). Phosphorylation serves as a primary regulatory mechanism that generally enhances FOXM1 activity, with its phosphorylation status being dynamically modulated throughout the cell cycle. Multiple kinases target serine, threonine, and tyrosine residues within the C-terminal transactivation domain of FOXM1, thereby fine-tuning its activity in a context- and phase-dependent manner [61].

Low levels of FOXM1 phosphorylation are characteristic of the period preceding the G1/S transition [62]. At this stage, FOXM1 predominantly exists as an inactive dimer, in which the N-terminal repressive domain of one monomer inhibits the C-terminal transactivation domain of the other [43]. Transition to an active state is facilitated by cell cycle-dependent kinase signaling, with the phosphatases CDC25A and CDC25B indirectly promoting FOXM1 activation through the activation of cyclin-dependent kinases (Figure 4) [63,64].

During the G1/S transition, CDK2–cyclin E complexes activate FOXM1 [65]. As cells progress through G1 phase, FOXM1 phosphorylation is further enhanced by CDK4 and CDK6 in association with cyclin D, promoting protein stabilization and protecting FOXM1 from proteasomal degradation [62].

Prior to mitotic entry, CDK1 and CDK2 in complex with cyclin A phosphorylate FOXM1 at specific threonine and serine residues (Thr596 and Ser678), thereby relieving its autoinhibitory conformation and enabling full transcriptional activation [43,47,66]. During the G2 phase, FOXM1 additionally associates with CDK1-cyclin B complexes, further promoting its activation in preparation for mitosis [67].

During mitosis, FOXM1 undergoes hyperphosphorylation mediated by polo-like kinase 1 (PLK1), which enhances its stability by protecting it from proteasomal degradation and ensuring proper mitotic progression (Figure 4) [68,69]. During this phase, FOXM1 also functionally interacts with NEK2 (Figure 4), and this interaction has been shown to promote the proliferation, migration, and invasion of osteosarcoma and esophageal squamous cell carcinoma cells [70,71]. Mitotic exit is accompanied by FOXM1 dephosphorylation mediated by the phosphatase PP2A, leading to attenuation of its activity [72].

In addition to cell cycle-dependent regulation, FOXM1 stability is modulated by the Wnt/β-catenin signaling pathway through phosphorylation-dependent mechanisms. Under basal conditions, the APC/Axin/GSK3 complex phosphorylates FOXM1 at Ser474, promoting FBXW7-mediated ubiquitination and proteasomal degradation (Figure 4). Activation of Wnt signaling inhibits this phosphorylation cascade and facilitates the recruitment of the deubiquitinase USP5, resulting in FOXM1 stabilization (Figure 4). Consequently, stabilized FOXM1 accumulates in the nucleus, where it cooperates with β-catenin and TCF4 to enhance the transcription of genes associated with cell proliferation (Figure 4) [73].

#### 3.2.5. Acetylation

FOXM1 transcriptional activity is also modulated by epigenetic regulators that influence its acetylation status. The transcriptional coactivators p300/CBP acetylate FOXM1 at lysine residues within its C-terminal domain, thereby enhancing its ability to activate target gene expression (Figure 4). Notably, FOXM1 acetylation levels increase during the S phase and remain elevated through mitosis, reflecting its role in cell cycle progression [74]. In contrast, class III histone deacetylases SIRT1 and SIRT2 counteract this modification by removing acetyl groups, leading to attenuation of FOXM1 transcriptional activity [74,75].

#### 3.2.6. Methylation

Methylation-dependent mechanisms further expand the regulatory landscape of FOXM1 and its associated transcriptional network. Under normoxic conditions, the methyltransferase SETD3 methylates FOXM1 and suppresses transcription of the vascular endothelial growth factor (*VEGF*) gene. In contrast, hypoxic conditions promote dissociation of the SETD3-FOXM1 complex from the *VEGF* promoter, thereby enabling VEGF expression [76]. FOXM1 also cooperates with epigenetic modifiers to regulate the expression of tumor suppressor genes. Its interaction with DNMT3B and RB1 (Figure 4) facilitates methylation of the *FOXO1* promoter leading to transcriptional repression of this tumor suppressor [77] and a consequent reduction in cyclin-dependent kinase inhibitor expression. Similarly, the FOXM1-DNMT3B-RB1 complex promotes methylation of the *GATA3* promoter, impairing normal epithelial differentiation and contributing to malignant transformation in breast tissue [78]. In addition, FOXM1 functionally interacts with the enhancer of zeste homolog 2 (EZH2), a key component of the polycomb repressive complex 2 (PRC2) (Figure 4). Although EZH2 classically mediates histone H3 methylation and transcriptional repression, its association with FOXM1 enables it to function as a scaffold protein, facilitating the coordinated regulation of target genes [79], including those involved in extracellular matrix remodeling, such as metalloproteinases [80].

Collectively, these regulatory mechanisms ensure tight spatiotemporal control of FOXM1 expression, stability, and transcriptional activity. The integration of signaling pathways, post-transcriptional regulators, and post-translational modifications enables rapid and context-dependent modulation of FOXM1 function. This regulatory flexibility allows FOXM1 to coordinate multiple transcriptional and signaling programs involved in tumor progression and cellular stress responses.

## 4. FOXM1 Involvement in Cellular Process Regulation

As a transcription factor, FOXM1 plays a central role in oncogenesis by orchestrating a broad range of cellular processes critical for tumor development and progression. Beyond its canonical role in transcriptional regulation, FOXM1 also exerts its effects through extensive protein–protein interactions, thereby integrating diverse signaling pathways and modulating multiple aspects of cellular function.

### 4.1. FOXM1 Transcriptional Activity

FOXM1 plays a pivotal role in cell cycle progression. It activates transcriptional programs required for the G1/S and G2/M transitions, as well as for DNA replication [81,82,83], spindle assembly, and checkpoint control [84,85,86]. Through the transcription activation of components of the SCF ubiquitin ligase complex, FOXM1 facilitates the degradation of cyclin-dependent kinase inhibitors, including p16, p27, and p21 [87,88]. Through these mechanisms, FOXM1 accelerates cell cycle progression, enhances proliferation capacity, and enables tumor cells to bypass DNA damage-induced cell cycle arrest.

Beyond its role in cell cycle regulation, FOXM1 contributes to cancer cell survival following genotoxic stress by promoting the expression of genes involved in multiple DNA repair pathways, including *RAD51*, *EXO1*, *BRCA1/2*, and others [89,90,91]. Through the coordinated activation of DNA repair machinery, elevated FOXM1 expression enhances the ability of tumor cells to resolve therapy-induced DNA damage, thereby contributing to the development of drug resistance [90].

In response to the increasing metabolic and oxygen demands during tumor growth, FOXM1 promotes angiogenesis through transcriptional activation of vascular growth factors including VEGF and PDGF [92,93]. Beyond promoting vascularization, FOXM1 also enhances metastatic dissemination by inducing epithelial–mesenchymal transition (EMT), migration, and invasion programmes. Mechanistically, FOXM1 activates EMT-associated transcription factors such as SNAIL and ZEB1, as well as downstream effectors, including the matrix-remodeling enzymes MMP2 and MMP9 [94,95,96,97]. These activities facilitate tumor cell invasion and adaptation to changing microenvironmental conditions.

FOXM1 is also required for the maintenance of cancer stem cell phenotypes through the activation of the expression of pluripotency-associated transcription factors such as *POU5F1* (OCT4), *NANOG*, and *SOX2* [98,99]. Due to their ability to initiate tumor growth and withstand therapeutic stress, cancer stem cells contribute to disease progression and relapse [100].

FOXM1 contributes to metabolic reprogramming by promoting aerobic glycolysis and lipid biosynthesis, thereby supporting tumor adaptation to metabolic stress. Mechanistically, FOXM1 induces the expression of pyruvate dehydrogenase kinase, facilitating a shift toward aerobic glycolysis [101]. It also regulates the expression of genes involved in lipid metabolism, including fatty acid synthase and acetyl-CoA carboxylase [102,103], enabling tumor cells to utilize fatty acids as an alternative energy source. In parallel, FOXM1 enhances glucose uptake and glycolytic flux by upregulating the expression of glucose transporters and glycolytic enzymes, leading to increased lactate production [102,104,105,106]. FOXM1 also enhances tumor cell survival under oxidative stress, primarily through the activation of transcriptional programs involved in reactive oxygen species detoxification, including superoxide dismutase 2 (SOD2), catalase (CAT), NRF2 (NFE2L2) and other related genes [107,108,109]. By reducing intracellular levels of reactive oxygen species, FOXM1 enhances cellular resistance to therapy-induced oxidative stress [110].

The multifaceted impact of FOXM1 on tumor cell survival is further underscored by its ability to regulate programmed cell death pathways. FOXM1 suppresses apoptosis by upregulating the anti-apoptotic proteins BCL-2 and MCL1 [111,112,113,114], as well as inhibitors of apoptosis such as XIAP and survivin [115,116], while downregulating the pro-apoptotic proteins BAX and BMF [114,117]. Additionally, FOXM1 suppresses anoikis, a form of apoptosis induced by the loss of extracellular matrix attachment. This function is particularly relevant during tumor dissemination, where interactions between platelets and circulating tumor cells activate FOXM1 via the PI3K/AKT signaling pathway, thereby promoting cell survival in the bloodstream [118,119]. FOXM1 also induces the expression of autophagy-related genes such as LC3 and beclin-1 [120,121]. Notably, FOXM1 levels increase in response to autophagy induction by nutrient deprivation or rapamycin treatment, whereas FOXM1 inhibition suppresses autophagic activity [120]. Given that autophagy supports tumor cell survival under stress conditions, FOXM1-mediated activation of this pathway is likely to contribute to the development of chemoresistance [122].

Finally, FOXM1 contributes directly to drug resistance by inducing the expression of ATP-binding cassette (ABC) transporters, including ABCB1, ABCC4, and ABCC5. These transporters facilitate drug efflux, thereby reducing intracellular drug accumulation [123,124,125].

Taken together, these findings demonstrate that FOXM1 regulates multiple interconnected programs involved in proliferation, stress adaptation, metabolic reprogramming, and resistance to cell death. Many of these canonical FOXM1 functions have been extensively discussed in previous reviews. However, emerging evidence indicates that FOXM1 activity is additionally shaped by extensive protein–protein interaction networks and intercellular communication mechanisms that remain comparatively underexplored. To further examine these noncanonical aspects of FOXM1 functions, the following section focuses on its interactome and protein–protein interaction networks.

### 4.2. FOXM1 Protein–Protein Interactions Mediating Its Functions

Beyond its canonical transcriptional activity, FOXM1 engages in extensive protein–protein interactions that critically modulate its functional output. In this section, we complement evidence from the literature with interactome data derived from the BioGRID database [43], thereby providing a more comprehensive view of FOXM1-associated regulatory networks.

#### 4.2.1. Interactions with Expression Regulators

Activated FOXM1 interacts with multiple transcriptional complexes and chromatin regulators, thereby shaping diverse gene expression programs. Its binding partners include key transcription factors such as MYC, BCL6, SP1, RELA, SMAD3, and HIF1A (Figure 4), highlighting the involvement of FOXM1 in the coordination of proliferation-associated, inflammatory, stress-response, and hypoxia-adaptive transcriptional programs [126,127,128,129,130,131]. FOXM1 also interacts with members of the nuclear factor I family (NFIA, NFIB, NFIC, and NFIX), which regulate cell proliferation and differentiation [132], as well as with interferon regulatory factor 7 (IRF7), a critical mediator of interferon signaling (Figure 4) [133].

In addition, FOXM1 can interact with transformation/transcription domain-associated protein (TRRAP), a member of the phosphatidylinositol 3-kinase-related kinase family (Figure 4), thereby potentially modulating multiple oncogenic and stress-responsive transcriptional programs [134]. Interactions with components of the TFIIIC complex and with the BANP protein further implicate FOXM1 in promoter recognition and the regulation of chromatin accessibility (Figure 4) [135,136].

#### 4.2.2. Nuclear Condensate Formation

FOXM1 can form transcriptional condensates at target gene promoters through its intrinsically disordered regions via liquid–liquid phase separation, providing an additional layer of regulatory control over its activity. The formation of these condensates is promoted by abnormal spindle-like microcephaly associated protein (ASPM), which enhances FOXM1 stability and transcriptional output. This process depends on the phosphorylation status of FOXM1 at Ser376 [137]. Phosphorylation of FOXM1 at Ser376 by AMP-activated protein kinase (AMPK) disrupts condensate formation, leading to cytosolic DNA accumulation and subsequent activation of innate immune signaling pathways [25]. The promyelocytic leukemia protein (PML) negatively regulates FOXM1 by sequestering it within PML nuclear bodies (Figure 4), thereby attenuating its transcriptional activity and shifting the regulatory balance toward FOXO3-mediated tumor suppression [138]. Collectively, these observations indicate that spatial organization within the nucleus represents an additional layer of FOXM1 regulation that dynamically modulates its transcriptional output in response to cellular conditions.

#### 4.2.3. Regulation of Cell Division

Cell cycle gene expression is controlled by the coordinated activity of the DREAM, RB1-E2F, and MuvB complexes [139,140]. The MuvB core complex, comprising LIN9, LIN37, and LIN54, functions as a context-dependent regulator capable of both activating and repressing transcription, depending on the stage of the cell cycle. MuvB associates with the DREAM and RB1–E2F complexes to repress the expression of G1/S phase genes. In contrast, upon entry into mitosis, MuvB transitions into an active transcriptional state through association with MYBL2, which facilitates the recruitment of FOXM1 to target promoters (Figure 4) [140]. While these interactions highlight the role of FOXM1 in transcriptional regulation, its function extends beyond the control of gene expression and involves the direct coordination of cell cycle-associated processes at the protein level.

In addition to its role in transcriptional regulation, FOXM1 also directly interacts with proteins involved in chromosome dynamics and cell division. These include kinesins such as KIF22 and KIF18A [141,142], as well as regulators of DNA replication (ORC2), chromosome condensation (SMC2), and cytokinesis (PRC1, ECT2) (Figure 4) [142]. Through these interactions, FOXM1 supports genome stability by protecting telomerase reverse transcriptase (TERT) from degradation through direct interaction (Figure 4) [143]. Furthermore, its association with telomeric repeat-binding factor 1 (TERF1) and histone H2B variants suggests a potential role in the regulation of telomere function and chromatin architecture (Figure 4) [144].

#### 4.2.4. Interactions with DNA Repair Factors

In addition to the transcriptional regulation of canonical DNA repair genes, FOXM1 directly interacts with multiple proteins involved in chromatin remodeling and DNA damage response signaling. For instance, the chromatin remodeling factor SMARC5A is recruited to sites of DNA damage, where it cooperates with FOXM1 to facilitate transcriptional recovery following genotoxic stress (Figure 4) [145]. In contrast, p53 can inhibit FOXM1 by preventing its recruitment to target gene promoters such as that of MELK, thereby attenuating FOXM1-driven transcriptional programs (Figure 4) [146]. DNA damage signaling pathways can also modulate FOXM1 activity through post-translational regulation. The checkpoint kinase CHEK2 phosphorylates FOXM1 in response to DNA damage, enhancing its stability and promoting the expression of DNA repair genes, including XRCC1 and BRCA2 (Figure 4) [147]. In a pathological context, FOXM1 interactions can contribute to therapy resistance. For example, in glioblastoma, the interaction between PARP3 and FOXM1 enhances FOXM1 transcriptional activity, thereby promoting resistance to radiotherapy (Figure 4) [148].

#### 4.2.5. Interactions with mRNA and Protein Metabolism Proteins

FOXM1 interacts with several proteins involved in RNA processing and translational regulation. These include splicing factors such as PRPF8, SNRNP200, SART1, and RBMX [142], as well as the RNA export factor ZC3H11A [149] and RNA helicases, including DHX29 and DDX52 (Figure 4) [150,151]. Such interactions suggest that FOXM1 may contribute to post-transcriptional regulation beyond its canonical role as a transcription factor. In addition, FOXM1 has been shown to interact with several aminoacyl-tRNA synthetases and tRNA-modifying enzymes, indicating a potential role in translation efficiency and tRNA stability (Figure 4) [142]. Furthermore, FOXM1 interacts with the molecular chaperones HSP90AA1 and DNAJC9 (Figure 4), which are involved in protein folding and the maintenance of genome stability [152,153]. Although the functional significance of many of these interactions remains insufficiently characterized, these findings suggest that FOXM1 may participate in broader post-transcriptional and translational regulatory networks than previously appreciated.

#### 4.2.6. FOXM1 in Intercellular Communication

In addition to its cell-intrinsic functions, FOXM1 also plays an important role in shaping an aggressive tumor microenvironment through intercellular communication. FOXM1 can be disseminated via extracellular vesicles, thereby influencing the behavior of neighboring cells. Tumor cells secrete exosomes enriched in FOXM1 which can be taken up by cells within the tumor microenvironment, including tumor-associated macrophages. Uptake of these vesicles increases FOXM1 levels in macrophages and promotes their polarization toward the M2 phenotype [154,155,156]. Mechanistically, FOXM1 directly binds to the promoter region of the *IDO1* gene, which encodes indoleamine 2,3-dioxygenase 1, thereby enhancing its transcription in macrophages. Activation of the FOXM1/IDO1 axis suppresses ferroptosis in macrophages, thereby promoting and maintaining M2 polarization [155]. M2-polarized macrophages suppress anti-tumor immune responses and support tumor progression. Accordingly, their accumulation within the tumor microenvironment is associated with poor clinical outcomes and resistance to therapy across multiple cancer types [156].

FOXM1 has also been implicated in the selective packaging of nucleic acids into extracellular vesicles. Specifically, FOXM1 was shown to interact with the autophagy protein LC3/MAP1LC3 in the nucleus, thereby enabling selective loading of FOXM1-associated chromatin DNA fragments into extracellular vesicles in human A549 lung cancer cells [157]. This mechanism involves the translocation of FOXM1-bound DNA regions, including telomeric DNA and the double homeobox 4 (*DUX4*) locus, from the nucleus to the cytoplasm, followed by their incorporation into extracellular vesicles. Importantly, mutational disruption of the FOXM1–LC3 interaction impaired the loading of these DNA fragments into extracellular vesicles, providing direct mechanistic evidence that FOXM1 may function not only as extracellular vesicle cargo but also as a selective cargo-specifying factor [157]. This finding suggests a previously unrecognized role for FOXM1 in mediating the intercellular transfer of genetic material.

While tumor cells actively remodel their surroundings, components of the tumor microenvironment can, in turn, modulate FOXM1 expression in tumor cells. For example, in ovarian cancer, adipose-derived stem cells residing in the peritoneum release exosomes into the ascitic fluid, which are subsequently taken up by tumor cells and lead to FOXM1 activation, thereby promoting disease progression. Although the molecular mediators responsible for FOXM1 activation have not been identified, these findings further support the bidirectional nature of FOXM1-dependent communication between tumor cells and the tumor microenvironment [158].

In an ovarian cancer model, FOXM1 protein levels in tumor cells increased following incubation with a therapy-induced secretome derived from dying tumor cells [5]. This observation may reflect either the direct transfer of FOXM1 protein via extracellular vesicles or the upregulation of FOXM1 expression in recipient cancer cells in response to secretome-associated signaling molecules.

**Figure 5:**
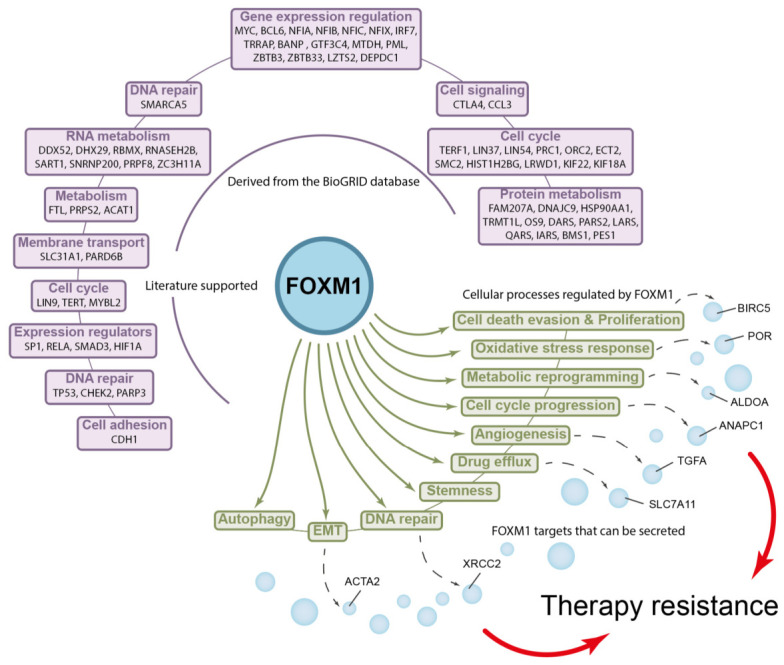
Integrated model of FOXM1-associated protein interaction and regulatory networks underlying therapy resistance. Literature-supported and BioGRID-derived FOXM1 interactors are classified according to their biological function (purple boxes), whereas downstream FOXM1-regulated processes and representative target genes are shown on the right (green boxes). Through coordinated regulation of proliferation, stress adaptation, metabolism, DNA repair, stemness, EMT, angiogenesis, and drug resistance programs (solid lines), FOXM1 promotes the acquisition of therapy resistance. In addition, selected FOXM1-regulated proteins identified in extracellular vesicle proteome datasets are indicated by dashed lines, suggesting that they may be transferred via extracellular vesicles between cells within the tumor microenvironment.

In addition to being transferred between cells, FOXM1 may also influence intercellular communication by regulating the composition of extracellular vesicles at the transcriptional level. To explore this possibility, we analyzed a list of FOXM1 target genes obtained from ChIP-seq experiments using the ChEA3 database [159] and integrated these data with vesicle-associated protein datasets from Vesiclepedia [160] and the Human Protein Atlas [161]. This integrative approach enabled the identification of FOXM1-regulated proteins with potential roles in extracellular signaling (Figure 5). Among these candidates were regulators of PI3K- and GTPase-associated signaling pathways, including PIK3R3 and SGK3. We also identified factors involved in apoptosis and proliferation such as TNFSF14 and TGFA, as well as proteins associated with cell cycle and DNA repair, including BIRC5, CKS1B, CKS2, and XRCC2. Additional candidates were linked to RNA processing (ADARB1 and SMU1), and metabolic reprogramming (ALDOA, AGL, RPIA, and GNPDA2). The complete list of FOXM1 targets identified through ChIP-seq analysis and their overlap with vesicle-associated proteins is provided in Supplementary Table S1.

Although the functional significance of many of these putative vesicle-associated FOXM1 targets remains to be experimentally validated, these findings suggest that FOXM1 may contribute to shaping the extracellular signaling landscape of the tumor microenvironment not only through direct intercellular transfer but also by regulating the molecular cargo of extracellular vesicles.

## 5. Pharmacological Inhibition of FOXM1 Activity

A growing body of evidence identifies FOXM1 as a key regulator of chemoresistance, with its inhibition significantly enhancing tumor cell sensitivity to conventional anticancer therapies [89,162]. These findings position FOXM1 as an attractive therapeutic target for overcoming treatment resistance.

To date, several strategies have been proposed to suppress FOXM1 activity, including small-molecule inhibitors, proton pump inhibitors, proteolysis-targeting chimeras (PROTACs), inhibitory peptides, aptamers, and proteasome inhibitors. Among these, several small-molecule compounds have been developed to directly target FOXM1, including synthetic inhibitors such as FDI-6 [163], XST-20 [164], and XST-119 [165], as well as thiazole antibiotics, including thiostrepton [166] and siomycin A [167]. These compounds interact with FOXM1 by binding to its DNA-binding domain and impairing its ability to activate the transcription of target genes. Despite promising preclinical results, their clinical translation remains limited by poor pharmacokinetic properties, insufficient selectivity, and off-target effects. For example, FDI-6 exhibits low systemic bioavailability in vivo [168]. Thiostrepton has been shown to inhibit FOXM1 and sensitize ovarian cancer cells to platinum-based drugs [89]; however, its lack of specificity, including its activity as a proteasome inhibitor [169], raises concerns regarding off-target effects and therapeutic selectivity.

Recent studies have identified FDA-approved proton pump inhibitors, including pantoprazole and rabeprazole, as potential FOXM1 inhibitors [170]. These compounds have been reported to bind the FOXM1 DNA-binding domain and suppress its transcriptional activity. However, the current evidence is largely limited to in silico and in vitro studies, and their therapeutic relevance in oncology remains to be established in vivo.

Alternative strategies focus on promoting FOXM1 degradation rather than directly inhibiting its transcriptional activity. Small molecules such as STL427944 and STL001 induce FOXM1 translocation from the nucleus to the cytoplasm, thereby facilitating its degradation via autophagy, although the precise molecular mechanisms underlying this process remain unclear [171,172]. Targeted protein degradation technologies represent a particularly promising avenue for FOXM1-directed therapy. PROTAC-based approaches enable the selective ubiquitination and degradation of target proteins and may therefore help overcome some of the challenges associated with targeting transcription factors [173]. Several FOXM1-directed PROTAC strategies have shown promising preclinical activity. For example, cell-penetrating peptide PROTACs effectively induce FOXM1 degradation, suppress tumor growth, and exhibit low toxicity in experimental models [174]. More recently, nanoplatform-based systems such as the self-assembling PROTAC prodrug NFTP have been developed to improve drug delivery, enhance bioavailability, increase tumor specificity, and reduce systemic toxicity [175].

Additional strategies focus on disrupting FOXM1-associated protein interactions. Peptides such as p19^ARF^ 26–44 [176] and M1-20 [177] interfere with FOXM1-associated complexes, thereby attenuating its transcriptional activity. Nucleic acid–based approaches, particularly DNA aptamers, have emerged as promising FOXM1 inhibitors [178]. These aptamers bind to the FOXM1 DNA-binding domain and prevent its interaction with target gene promoters. Compared with conventional small-molecule inhibitors, aptamers offer improved specificity and favorable opportunities for chemical optimization [178]. For instance, phosphorothioate modifications can enhance resistance to nuclease-mediated degradation and improve stability in the bloodstream [179].

Proteasome inhibitors, such as bortezomib and MG132, can indirectly suppress FOXM1 activity through the modulation of protein homeostasis. By inhibiting proteasomal degradation, these agents promote the accumulation of heat shock proteins, including HSP70, which function as endogenous inhibitors of FOXM1 [180,181]. Several newly developed synthetic FOXM1 inhibitors, including NB-55, NB-73, and NB-115, have demonstrated improved pharmacokinetic properties compared with earlier FOXM1 inhibitors [168]. However, these agents lack strict specificity and can also target other members of the FOX transcription factor family, underscoring the persistent challenge of achieving selective FOXM1 inhibition [182].

Despite the diversity of these approaches, no FOXM1-targeting agent has yet been approved for clinical use, and no clinical trials are currently underway [183]. This reflects several fundamental challenges. First, as a transcription factor, FOXM1 lacks a well-defined catalytic pocket, making the development of highly specific small-molecule inhibitors inherently difficult [184]. Second, many currently available compounds exhibit limited selectivity and are associated with significant off-target effects [168,169,182]. Third, the inhibition of FOXM1 can be functionally compensated by the activation of alternative oncogenic pathways, reducing the efficacy of monotherapeutic approaches [185].

Potential toxicity further complicates the therapeutic targeting of FOXM1 [186,187]. Although its expression is relatively low in most differentiated tissues (Figure 1) [188], FOXM1 remains active in highly proliferative compartments, such as the bone marrow, testis, and thymus, raising concerns regarding on-target adverse effects. Moreover, given its role in normal tissue homeostasis, FOXM1 inhibition may impair regenerative processes and tissue repair [189]. Nonetheless, the markedly higher expression of FOXM1 in tumor cells compared with normal tissues suggests the existence of a potential therapeutic window that could be exploited under carefully controlled conditions [9].

In light of these limitations, combination strategies are likely to represent the most promising avenue FOXM1-targeted therapy [190]. Integrating FOXM1 inhibition with conventional chemotherapeutic agents, BCL2 inhibitors, or cell cycle regulators has demonstrated enhanced antitumor efficacy in preclinical models. Future efforts should, therefore, focus on improving the specificity and pharmacological properties of FOXM1 inhibitors, optimizing delivery platforms, and developing rational combination regimens. Targeting downstream effectors or FOXM1-dependent signaling networks may provide an alternative strategy to circumvent the challenges associated with direct FOXM1 inhibition [191].

## 6. Conclusions

FOXM1 has emerged as a central regulator of tumor cell adaptation to therapeutic stress, orchestrating a broad spectrum of processes that collectively drive cancer progression and treatment resistance. Through its roles in cell cycle control, DNA damage repair, metabolic reprogramming, and stress response, FOXM1 enables tumor cells to maintain proliferative capacity and survive under the adverse conditions induced by anticancer therapies.

Importantly, accumulating evidence indicates that FOXM1 function extends beyond cell-intrinsic mechanisms. As highlighted in this review, FOXM1 participates in complex protein–protein interaction networks and regulates multiple layers of gene expression, thereby integrating diverse intracellular signaling pathways. In addition, FOXM1 contributes to intercellular communication within the tumor microenvironment through its involvement in extracellular vesicle-mediated signaling and responses to therapy-induced secretomes. Collectively, the evidence discussed in this review supports the view that FOXM1 should be considered not simply a regulator of individual oncogenic pathways, but a systems-level integrator that coordinates intracellular stress adaptation and microenvironment-driven mechanisms of therapy resistance. Despite its strong therapeutic potential, targeting FOXM1 remains challenging. The lack of well-defined druggable domains, functional redundancy within oncogenic signaling networks, and compensatory activation of alternative pathways limit the efficacy of current FOXM1-targeting strategies. Furthermore, given the role of FOXM1 in normal proliferative tissues, careful consideration of potential toxicity is required when developing therapeutic approaches.

Future studies should therefore focus on a more comprehensive characterization of FOXM1-centered regulatory networks, including its interactome and role in intercellular signaling. Particular attention should be paid to the identification of context-specific dependencies and vulnerabilities that may enable selective targeting of FOXM1-driven pathways. In this regard, combination strategies integrating FOXM1 inhibition with conventional therapies, targeted agents, or modulators of the tumor microenvironment represent a particularly promising direction. In addition, targeting downstream effectors or FOXM1-dependent signaling circuits may provide an alternative approach for overcoming the challenges associated with direct FOXM1 inhibition.

In summary, FOXM1 represents a critical node in the regulatory architecture of tumor adaptation and therapy resistance. A deeper understanding of its multifaceted functions may facilitate the development of more effective therapeutic strategies and ultimately improve clinical outcomes for patients with cancer.

## Data Availability

The data used in this study are available in the ChEA3, GEPIA2, Vesiclepedia, and Human Protein Atlas databases. These data were derived from the following resources available in the following public domains: https://maayanlab.cloud/chea3/ (ChEA3, accessed on 20 April 2022), http://gepia2.cancer-pku.cn/ (GEPIA2, accessed on 5 April 2026), http://www.microvesicles.org/ (Vesiclepedia, accessed on 5 April 2026), and https://www.proteinatlas.org/ (Human Protein Atlas, accessed on 2 December 2022).

## Supplementary Materials

The following supporting information can be downloaded at https://www.mdpi.com/article/10.3390/ijms27125265/s1.
